# Predictors of lower exercise capacity in patients with cancer

**DOI:** 10.1038/s41598-023-41390-0

**Published:** 2023-09-08

**Authors:** Ruben Evertz, Christine Diehl, Katharina Gödde, Miroslava Valentova, Tania Garfias-Veitl, Tobias R. Overbeck, Friederike Braulke, Alessia Lena, Sara Hadzibegovic, Annalen Bleckmann, Ulrich Keller, Ulf Landmesser, Alexander O. König, Gerd Hasenfuss, Andreas Schuster, Markus S. Anker, Stephan von Haehling

**Affiliations:** 1 Department of Cardiology and Pneumology, University of Göttingen Medical Center (UMG), Georg-August-University Göttingen, Robert-Koch-Str. 40, 37075 Göttingen, Germany; 2 German Center for Cardiovascular Research (DZHK), Partner Site Göttingen, Göttingen, Germany; 3 Department of Paediatrics, University of Göttingen Medical Center (UMG), 37075 Göttingen, Germany; 4 Department of Haematology and Medical Oncology, University of Göttingen Medical Center (UMG), 37075 Göttingen, Germany; 5 Comprehensive Cancer Center G-CCC, Medical Center (UMG), University of Göttingen, 37075 Göttingen, Germany; 6 Department of Gastroenterology, University of Göttingen Medical Center (UMG), 37075 Göttingen, Germany; 7 Charité – Universitätsmedizin Berlin, Corporate Member of Freie Universität Berlin and Humboldt-Universität zu Berlin, Charitéplatz 1, 10117 Berlin, Germany; 8 Deutsches Herzzentrum der Charité, Klinik für Kardiologie, Angiologie und Intensivmedizin, Hindenburgdamm 30, 12203 Berlin, Germany; 9 German Center for Cardiovascular Research (DZHK), Partner Site Berlin, Berlin, Germany; 10 Berlin Institute of Health Center for Regenerative Therapies (BCRT), 13353 Berlin, Germany; 11 West German Cancer Center, University Hospital Münster, 48149 Münster, Germany; 12 Department of Medicine A for Hematology, Oncology and Pneumology, University Hospital Münster, 48149 Muenster, Germany; 13 Department of Hematology, Oncology and Cancer Immunology, Charité – Universitätsmedizin Berlin, Campus Benjamin Franklin, 12203 Berlin, Germany; 14 German Cancer Consortium (DKTK) & German Cancer Research Center (DKFZ), 69120 Heidelberg, Germany; 15 Berlin Institute of Health (BIH), 13353 Berlin, Germany; 16 Max-Delbrück-Center for Molecular Medicine, 13125 Berlin, Germany

**Keywords:** Cardiology, Oncology

## Abstract

Maintaining cancer patients’ exercise capacity and therefore patients’ ability to live a self-determined life is of huge importance, but little is known about major determinants. We sought to identify determinants of exercise capacity in patients with a broad spectrum of cancer types, who were already receiving cancer treatment or about to commence such therapy. Exercise capacity was assessed in 253 consecutive patients mostly suffering from advanced cancer using the 6-min walk test (6-MWT). All patients underwent echocardiography, physical examination, resting electrocardiogram, hand grip strength (HGS) measurement, and laboratory assessments. Patients were divided into two groups according to the median distance in the 6-MWT (459 m). Patients with lower exercise capacity were older, had significantly lower HGS and haemoglobin and higher values of high sensitive (hs) Troponin T and NT-proBNP (all *p* < 0.05). Whilst the co-morbidity burden was significantly higher in this group, no differences were detected for sex, body mass index, tumor type, or cachexia (all *p* > 0.2). Using multivariable logistic regression, we found that the presence of anaemia (odds ratio (OR) 6.172, 95% confidence interval (CI) 1.401–27.201, *p* = 0.016) as well as an increase in hs Troponin T (OR 3.077, 95% CI 1.202–5.301, *p* = 0.019) remained independent predictors of impaired exercise capacity. Increasing HGS was associated with a reduced risk of a lower exercise capacity (OR 0.896, 95% CI 0.813–0.987, *p* = 0.026). Screening patients for elevated hs troponin levels as well as reduced HGS may help to identify patients at risk of lower exercise capacity during cancer treatment.

## Introduction

In 2018, the World Health Organization (WHO) reported 18.1 million new cases of cancer worldwide, with lung, breast, and prostate cancer being the most frequent entities^1^. Cardiotoxicity of anti-cancer treatments has become an increasingly important aspect in the care of such patients, leading to the involvement of cardiologists and the deployment of cardio-oncology services in comprehensive cancer centers in recent years^2^.

Cardio-oncology, however, involves more aspects than the treatment of cardiotoxic adverse events, it also covers cancer development in patients with manifest cardiac diseases like heart failure or other cardiovascular diseases in patients diagnosed with cancer^3,4^. Close interaction between the two fields is important, because patients are not only threatened by high mortality rates, but also by reduced exercise capacity and consequently impaired quality of life (QoL)^5,6^. Current evidence also suggests an interplay between cardiovascular and metabolic parameters and QoL in patients with cancer^7^.

Identifying patients at risk of diminishing exercise capacity and the identification of early interventional approaches may be a pivotal aspect in the care of affected patients^8^. Since similar problems are observed in patients with chronic heart failure, we aimed to assess cancer patients’ exercise capacity from a cardiology perspective. Even though this point is receiving increasing attention in recent years, advanced cardiovascular investigations do still not always belong to the routine clinical care among patients with cancer in general or in those scheduled to undergo systemic therapy.

Clinical trials in oncology are mainly driven by endpoints that target patients’ survival. Whilst this is clearly important, many patients in the clinical setting ask questions about maintaining mobility and QoL. Cardio-oncology is devoted to understanding the pathophysiological changes in the cardiovascular system in patients with cancer, offering an intriguing approach to improving cardiovascular function and possibly survival.

Parameters of interest have so far included left ventricular ejection fraction (LVEF), heart rate, blood pressure, heart rate variability, cardiac biomarkers, and strain imaging. Particularly LVEF has been in the focus of attention when cardiotoxic reactions are being assessed. Limited information is available on exercise capacity as measured using typical tests usually performed in clinical trials in cardiology. These include tests like spiroergometry (cardiopulmonary exercise testing) or the frequently performed 6-min walk test (6-MWT). Whilst spiroergometry allows detection of maximal and submaximal exercise capacity, the 6-MWT allows reliable assessment of everyday exercise performance.

Data from patients with colorectal cancer indicate that exercise capacity is impaired already in chemotherapy-naïve patients, but the start of respective therapies worsens this development^9^. Recent analyses using available treatment data from patients with heart failure have highlighted the immense difficulties to improve exercise capacity with the most robust data supporting therapeutic decrease of high heart rate values and the treatment of iron deficiency^10^. We sought to identify risk factors for the development of impaired exercise capacity in a mixed cohort of patients with cancer from different etiologies in order to counteract the deterioration in physical performance.

## Methods

We prospectively enrolled 253 cancer patients between November 2017 and July 2019 at the University Medical Center Göttingen and the Charité Medical School, Campus Benjamin Franklin, Berlin, both in Germany. Only patients with histologically confirmed cancer that were ≥ 18 years of age were included in this study.

The following criteria were defined as reasons for exclusion: (1) clinical signs of an acute infection or antibiotic treatment due to an infection, (2) relevant cardiovascular disease (e.g. coronary artery disease, prior myocardial infarction, or left ventricular ejection fraction (LVEF) < 50%, atrial fibrillation), (3) severe chronic obstructive pulmonary disease, defined as Global Initiative for Chronic Obstructive Lung Disease (GOLD) stage > II (except in lung cancer patients all GOLD stages were allowed), (4) and any other cancer diagnosis in the 5 years preceding enrolment^11^.

The inclusion and exclusion criteria result in a heterogenous cancer patient population and as a result cancer specific therapy varies as well. However, this approach allows an overview of general principles yielding reduced exercise performance measured by the 6-MWT, which are not restricted to a specific cancer type or a specific cancer therapy. Due to the all-comer status of the patients included in this analysis, most participants were already receiving cancer treatment or were about to commence such therapy.

All participants underwent a battery of tests, which included the acquisition of the patient’s medical history, a physical examination, a resting electrocardiogram (ECG), transthoracic echocardiography, blood collection as well as a 6-MWT and hand grip strength (HGS) assessment.

### Blood collection

Full blood count and clinical chemistry parameters were analyzed from a venous blood draw by the local laboratory. High sensitive (hs) Troponin T and N-terminal pro-B-type natriuretic peptide (NT-proBNP) were assessed using assays provided by Roche Germany Holding GmbH (Grenzach-Wyhlen, Germany).

### Cardiovascular assessment

Following the recording of the medical history, a detailed physical examination including measurement of body weight was performed. The blood pressure of all patients was assessed in a sitting position in a quiet environment after a rest of at least 5 min according to current guidelines^12^. Three measurements were performed 2 min apart using a Boso Medicus electronical sphygmomanometer (Bosch + Sohn GmbH und Co.KG, Juningen, Germany). Results were recorded as an average of the last two blood pressure readings. A 12 lead-electrocardiogram was recorded in a supine position using a MAC™ 3500 Resting ECG System (GE Healthcare, Chicago, Illinois, United States). Additionally, 24-h holter ECG data were acquired using a DMS300-4L recorder (DMS-Service, Los Angeles, California, United States). Echocardiography was performed with Vivid E9 ultrasound device (GE Healthcare, Chicago, Illinois, United States) to exclude a reduced ejection fraction as well as severe valve diseases.

Exercise capacity was evaluated by the 6-MWT, which was performed according to standard protocol^13^. Furthermore, physical strength was evaluated by measuring HGS. HGS measurements were performed in a sitting position using a Jamar® Plus + Digital Hand Dynamometer (Performance Health Holding, Inc., Warrenville, Illinois USA). While the elbow was flexed at 90°, shoulder and wrist were in a neutral position (0 degrees). HGS was evaluated in both hands, and the average of 3 tests of the stronger hand noted. Cachexia was defined as a weight loss of at least 5% of body weight within the last 12 month or a combination of 2% weight loss and a body mass index (BMI) < 20 kg/m^2^.

### Statistical analysis

Statistical analysis was performed using the Statistical Package for the Social Sciences (SPSS) version 26 for Windows (International Business Machines Corporation, Armonk, New York, USA). Normal distribution was tested using the Shapiro–Wilk test. Parametric data are expressed as mean ± standard deviation. Non-parametric data were compared using Mann–Whitney U and Kruskal–Wallis tests as appropriate and expressed as median an interquartile range. For between-group comparisons in parametric data t- or analysis of variance (ANOVA) testing was performed as appropriate. For binary variables the intergroup comparison was performed using the chi^2^ test. Simple regression was used to analyze first line associations between continuous variables. Univariable and multivariable logistic regression models were used to identify clinical determinants of exercise capacity in patients with advanced cancer and expressed as odds ratio and 95% confidence interval (CI). A *p*-value < 0.05 was considered statistically significant. All significant univariable parameters as well as creatinine and sex were included in the multivariable logistic regression model.

### Institutional Review Board statement

The study was conducted according to the guidelines of the Declaration of Helsinki, and approved by the local ethic committee of the University Medical Centre Göttingen (approval code 22/8/17; approval date 13.09.2017).

### Informed consent statement

Informed consent was obtained from all subjects involved in the study.

## Results

### Study population characteristics

**Figure 1:**
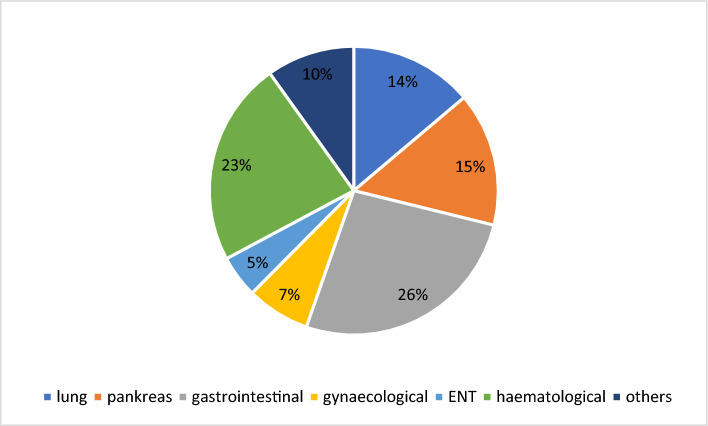
Tumor type distribution in the population under study. ENT, ear-nose and thorat.

A total 253 patients were included. Patients’ characteristics are displayed in Table 1: 57.7% of the study cohort were male. Mean age was 60.4 ± 12.5 years with age ranging from 20 to 83 years. Whilst most patients were diagnosed with a solid tumor (77.1%), only a minority had haematological malignancies (22.9%) (Fig. 1). Most of the patients were diagnosed with advanced disease stages: 89.4% patients were classified as UICC stadium ≥ III and 56.4% as Ann-Arbor-Stadium ≥ III. Follow-up was censored in February 2020 and the mean follow-up period was 12.1 ± 6.2 months. Until this point, a total of 84 (33.2%) patients had died. Mean body-mass-index (BMI) was 25.4 ± 4.6 kg/m^2^ and ranged from 14.9 to 41.6 kg/m^2^. The most common co-morbidities were anaemia (61.7%)—defined as a haemoglobin value < 13 g/dl in male and < 12 g/dl in female—followed by cachexia (46.6%) and hypertension (43.5%). Dyspnoea of any cause was reported in 30.4%, current smoking status in 15.0% and diabetes mellitus in 11.5% of patients.

**Table 1:** Baseline characteristics of the study population.

	Study population (n = 253)		6-MWT < median (n = 126)	6-MWT ≥ median (n = 127)	*p*-values
Demographic data					
Age (years)	60.4 ± 12.5 (20.0–83.0)		63.5 ± 12.7 (20–83)	57.3 ± 11.6 (23–81)	** < 0.001**
Sex (male)	146 (57.7%)		68 (54.0%)	78 (61.4%)	0.23
Height (m)	1.72 ± 0.09 (1.51–1.98)		1.70 ± 0.09 (1.51–1.90)	1.75 ± 0.09 (1.56–1.98)	** < 0.001**
Weight (kg)	76.2 ± 16.9 (42.0–150.0)		75.5 ± 17.9 (42.0–150.0)	77.0 ± 15.8 (43.0–120.3)	0.68
BMI (kg/m^2^)	25.4 ± 4.6 (14.9–41.6)		25.84 ± 4.9 (14.9–41.6)	25.0 ± 4.3 (16.5–36.3)	0.25
Haemato-oncology data					
Solid tumor	195 (77.1%)		95 (75.4%)	100 (78.7%)	0.53
UICC stadium	I = 4 (2.1%)	II = 17 (8.9%)			
	III = 26 (13.5%)	IV = 148 (75.9%)			
Haematological neoplasia	58 (22.9%)		31 (24.6%)	27 (21.3%)	0.53
Ann-Arbor stadium	I = 14 (25.5%)	II = 10 (18.2%)			
	III = 14 (25.5%)	IV = 17 (30.9%)			
Currently alive	169 (66.8%)		78 (61.9%)	91 (71.7%)	0.1
Co-morbidities					
Cachexia	118 (46.6%)		61 (48.4%)	57 (45.6%)	0.66
Hypertension	110 (43.5%)		73 (57.9%)	37 (29.1%)	** < 0.001**
Anaemia	156 (61.7%)		95 (75.4%)	61 (48%)	** < 0.001**
Diabetes mellitus	29 (11.5%)		20 (16.7%)	9 (7.7%)	**0.035**
Current smoker	38 (15%)		18 (22.2%)	20 (26.3%)	0.55
Dyspnoea (NYHA class ≥ II)	77 (30.4%)		49 (57.0%)	28 (29.5%)	** < 0.001**

Data are expressed as number (percentage) or mean ± standard deviation (minimum and maximum) irrespective of normal distribution. BMI, Body mass index; kg, kilogram; m, meters; NYHA, New York Heart Association; UICC, Union for International Cancer Control.

Significant values are in bold.

### Exercise capacity

**Figure 2:**
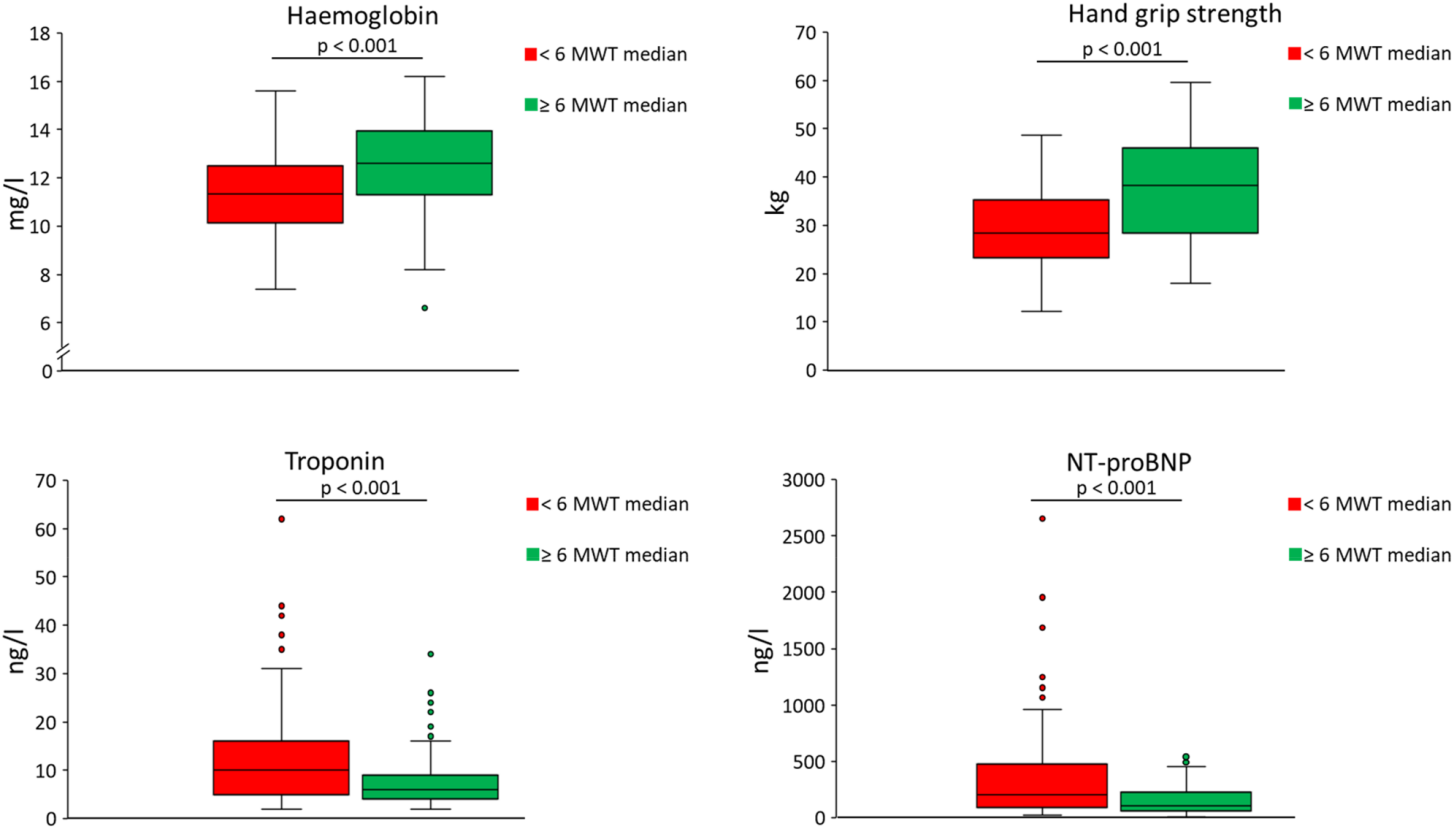
Intergroup comparison of selected laboratory measurements as well as Hand grip strength. 6-MWT: 6 min walk test.

Patients were divided into two groups based on the median distance in the 6-MWT (459 m). Patients with lower exercise capacity were older and had higher values of serum hs Troponin T and NT-proBNP (all *p* < 0.05). In this group, the co-morbidity burden was significantly higher with regards to anaemia (*p* < 0.001), hypertension (*p* < 0.001), and diabetes mellitus (*p* = 0.035). Patients with lower exercise capacity were significantly more likely to report dyspnoea on exertion (*p* = 0.003). No significant differences between the groups with lower *vs.* higher exercise capacity were found for sex (*p* = 0.23), BMI (*p* = 0.25), tumor type (*p* = 0.53), survival (*p* = 0.1), presence of cachexia (*p* = 0.66) and current smoking status (*p* = 0.55) (Table 1). Haemoglobin, transferrin, albumin, and sodium values were all significantly lower in patients with reduced exercise capacity (6-MWT below median, all *p* < 0.05). All laboratory findings are summarized in Table 2. Whilst there were no significant differences in LVEF between the two groups, heart rate, and diastolic blood pressure (all *p* > 0.05), systolic blood pressure (*p* = 0.007) was slightly lower and the HGS higher in patients with higher exercise capacity (*p* < 0.0001) (Table 3 and Fig. 2).

**Table 2:** Results of the laboratory measurements.

	Study population (n = 253)	6-MWT < median (n = 126)		6-MWT ≥ median (n = 127)	*p*-values	
Blood count						
Haemoglobin (g/dl)	11.98 ± 1.91 (6.60–16.20)	11.38 ± 1.82 (7.40–15.60)	12.58 ± 1.81 (6.60–16.20)			** < 0.001**
Haematocrit (%)	35.7 ± 5.4 (18.6–48.6)	33.98 ± 5.2 (22.3–45.7)	37.33 ± 5.1 (18.6–48.6)			** < 0.001**
Erythrocytes(× 10^6^/µl)	3.99 ± 0.70 (2.20–5.54)	3.75 ± 0.66 (2.27–5.20)	4.22 ± 0.65 (2.20–5.54)			** < 0.001**
Platelets (× 10^3^/µl)	236 (171.5–311.5)	238.5 (163.5–336.3)	233 (174–297)			0.26
Leucocytes (× 10^3^/µl)	5.82 (4.30–8.02)	5.82 (4.45–7.93)	5.82 (4.26–8.25)			0.64
Clinical chemistry panel						
Sodium (mmol/l)	140 (138–142)	139 (137–142)	140 (139–141)			**0.026**
Potassium (mmol/l)	4.15 (3.8–4.4)	4.10 (3.8–4.5)	4.2 (3.9–4.4)			0.65
Iron (µmol/l)	12.4 (8.5–18.8)	11.30 (8.25–16.10)	13.30 (8.60–19.25)			0.07
Transferrin (g/l)	2.29 (2.02–2.62)	2.22 (1.95–2.53)	2.41 (2.09–2.69)			**0.008**
Transferrin saturation (%)	21 (15.0–32.6)	21 (15.0–33.0)	21 (15.0–32.3)			0.67
Ferritin (µg/l)	203 (86.5–388.95)	210 (89.8–464.65)	192 (83.5–324.83)			0.44
Creatinine (mg/dl)	0.79 (0.68–0.95)	0.77 (067–0.95)	0.81 (0.70–0.95)			0.34
Albumin (g/l)	38 (35–41)	37 (34.0–40.0)	39.6 (36.0–41.4)			** < 0.001**
CRP (mg/l)	5.9 (1.90–14.75)	7.9 (2.65–16.20)	4.1 (1.33–10.65)			0.52
Cardiac biomarkers						
Troponin T (ng/l)	7.5 (4.0–13.0)	10.0 (5.0–16.0)	6.0 (4.0–9.0)			** < 0.001**
NT-pro-BNP (ng/l)	148.5 (65.0–308.0)	200.0 (87.0–481.0)	101.0 (61.0–226.5)			** < 0.001**

Normal distributed data are expressed as mean ± standard deviation and minimum and maximum values. Non parametric data are expressed as median and interquartile range. CRP, C-reactive protein; dl, deciliter; g, gram; l, liter; mg, milligram; ng, nanogram; µl, microliter.

Significant values are in bold.

**Table 3:** Instrument based diagnostic of the study population.

	Study population (n = 253)	6-MWT < median (n = 126)	6-MWT ≥ median (n = 127)	*p*-values	Effect size (Cohen’s d)
LVEF (%)	60 (56–67)	62 (56–67)	60 (56–67)	0.63	
Resting heart rate (bpm)	71 (64–83)	72 (43–84)	71 (63–81)	0.34	
BP systolic (mmHg)	126 (115–140)	130 (120–142)	125 (115–134)	**0.007**	
BP diastolic (mmHg)	80 (70–85)	80 (72–88)	80 (70–83)	0.08	
6-MWT (meters)	459 (380.5–508.0)	380.5 (327.75–426.0)	507.0 (478.0–562.0)	** < 0.001**	
HGS (kg)	33.29 ± 10.43 (12.07–59.6)	28.86 ± 8.05 (12.07–48.6) (n = 79)	38.02 ± 10.64 (17.9–59.6) (n = 74)	** < 0.001**	**0.97**

All data were not normal distributed and therefore expressed as median and interquartile range. BP, blood pressure; bpm, beats per minute; HGS, hand grip strength; LVEF, left ventricular ejection fraction; 6-MWT, six minute walk test; kg, kilogram; mmHg: millimetre Mercury.

Significant values are in bold.

### Predictors of lower exercise capacity

**Figure 3:**
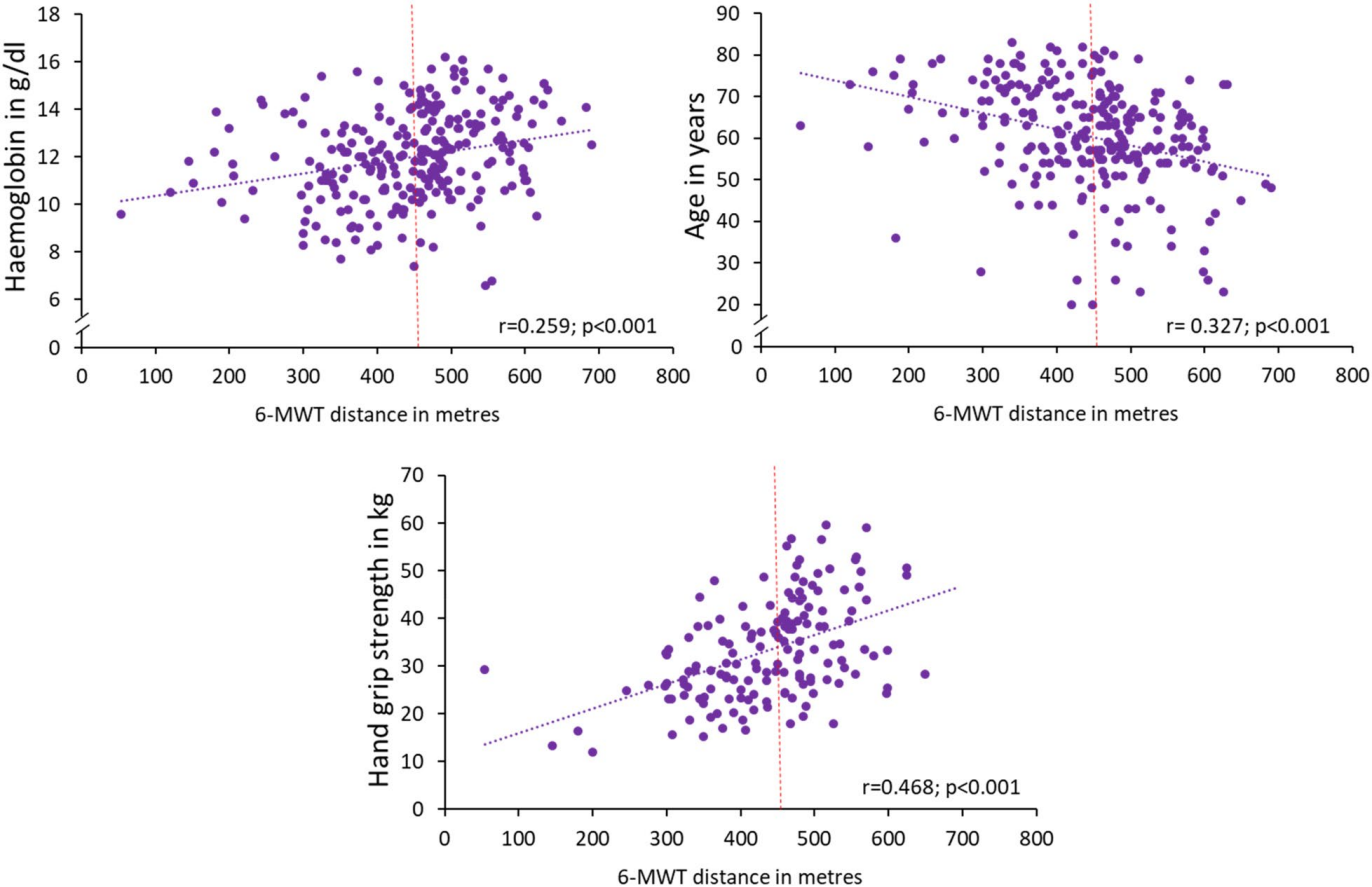
Linear Regression models illustrating the impact of haemoglobin, age and hand grip strength on the distance reached in the 6-MWT. The median of the 6-MWT is marked as dotted red line. 6-MWT: 6 min walk test.

Using univariable logistic regression, we found that hs Troponin T, NT-proBNP, age, history of hypertension, anaemia, diabetes mellitus, and dyspnoea all predicted lower exercise capacity (all *p* < 0.04). The same was true for haemoglobin and HGS in which decreasing values were associated with lower exercise capacity (Table 4, Fig. 3) (all *p* < 0.001). In addition, the presence of a hypalbuminaemia was associated with a lower exercise capacity in the univariable logistic regression model (*p* = 0.009). After multivariable adjustment, hs troponin T, anaemia, history of hypertension, and HGS all remained independent predictors of impaired exercise capacity (Table 4). NT-proBNP lost its predictive power after multivariable adjustment.

**Table 4:** Illustrating regression models for predicting a reduced exercise capacity defined as 6-MWT below median.

Variable	Odds ratio (95% CI)	*p*-values
Univariable models		
Age (per 1 year increase)	1.044 (1.021–1.067)	** < 0.001**
Sex (male)	0.737 (0.447–1.215)	0.23
Solid tumor (present)	0.827 (0.460–1.489)	0.25
BMI (per 1 kg/m^2^ increase)	1.040 (0.986–1.098)	0.15
Night sweat (present)	1.283 (0.699–2.356)	0.42
Cachexia (present)	1.120 (0.682–1.838)	0.66
Hypertension (present)	3.350 (1.990–5.642)	** < 0.001**
Anaemia (present)	3.316 (1.943–5.658)	** < 0.001**
Diabetes mellitus (present)	2.400 (1.044–5.517)	**0.039**
Current smoker (present)	0.800 (0.385–1.663)	0.55
Dyspnoea (present) (NYHA class ≥ II)	3.169 (1.715–5.854)	** < 0.001**
Iron deficiency (present)	1.123 (0.670–1.882))	0.66
Haemoglobin (per 1 g/dl increase)	0.695 (0.599–0.807)	** < 0.001**
Platelets (per 1 unit increase)	1.000 (1.000–1.002)	0.09
Leucocytes (per 1 unit increase)	1.036 (0.985–1.089)	0.17
Ferritin (per 1 µg/l increase)	1.000 (1.000–1.000)	0.71
TSAT (per 1 unit increase)	0.998 (0.985–1.012)	0.83
Hypalbuminaemia (present)	2.296 (1.228–4.292)	**0.009**
Log Troponin T (per 1 SD increase)	2.041 (1.429–2.917)	** < 0.001**
Log NT-proBNP (per 1 SD increase)	1.759 (1.340–2.309)	** < 0.001**
Log Creatinin (per 1 SD increase)	0.361 (0.042–3.102)	0.353
LVEF (per 1 unit increase)	1.004 (0.963–1.047)	0.84
Heart rate (per 1 bpm increase)	1.011 (0.991–1.031)	0.29
HGS (per 1 kg increase)	0.903 (0.868–0.940)	** < 0.001**
Multivariable model*		
Anaemia (present)	6.172 (1.401–27.201)	**0.016**
Hypertension (present)	8.164 (1.771–37.635)	**0.007**
HGS (per 1 kg increase)	0.896 (0.813–0.987)	**0.026**
Log Troponin T (per 1 SD increase)	3.077 (1.202–5.301)	**0.019**
Log creatinine (per 1 SD increase)	0.001 (0.000–0.037)	**0.006**

Expressed as Odds ratio and 95% confidence interval.

Significant values are in bold.

BMI, Body mass index; BP, blood pressure; bpm, beats per minute; CRP, C reactive protein; dl, deciliter; g, gram; HGS, hand grip strength; kg, kilogram; l, liter; LVEF, left ventricular ejection fraction; m, meter; NT-proBNP, N-terminal pro-B-type natriuretic peptide; NYHA, New York Heart Association; SD, standard deviation; TSAT, transferrin saturation; µg, microgram.

*Adjusted for: Age, sex (male), Hypertension, Anaemia, Diabetes mellitus, Dyspnoea (NYHA class ≥ II), Hypalbuminaemia, log Troponin, log NT-proBNP, HGS, log Creatinine.

## Discussion

Our data show that several factors are associated with reduced exercise capacity in patients with cancer. After multivariable adjustment, the presence of anaemia, increasing levels of hs troponin T and decreasing values in HGS remained significant predictors of impaired exercise capacity.

In clinical practice, reduced exercise capacity is reported by many patients with cancer, and this finding negatively impacts activity of daily living in cancer survivors^14^. In turn, reduced physical activity negatively impacts patients’ QoL and the so-called hard endpoints like cancer mortality and cancer recurrence^15^. In contrast, it is well known that being physically active is beneficial with regards to better physical and social functioning and also to coping with cancer and treatment-related side effects^16^.

Unfortunately, the number of studies trying to identify risk factors for reduced exercise capacity in patients with cancer remains limited. Miller et al*.* identified age, higher body fat content, methotrexate exposure and extremes of LV mass as predictors of lower exercise capacity in long-term paediatric cancer survivors measured as peak oxygen consumption (peak VO_2_). In line with the findings of our study, NT-proBNP failed as a useful predictor^17^. In contrast to the aforementioned study, our study found hs Troponin T to be a predictor of reduced exercise capacity in oncological patients even after multivariable adjustment. This is in line with data published by deFilippi et al*.* who found an association between troponin levels, exercise capacity and the risk of developing heart failure^18^. Interestingly, increasing troponin levels were also described as an independent predictor of mortality in the elderly population^19^. The same was found for respiratory diseases including COVID-19, renal disease and in patients suffering from coronary heart disease as well as from heart failure^20–24^. In the oncological setting, cardiac biomarkers, especially B-type natriuretic peptide as well as troponin, are usually used to detect cardiac side effects of cancer treatment or even the cancer itself^25^.

In recent years, the interest in cardiac biomarkers has increased in oncological patients. Indeed, Cardinale et al*.* were able to show that troponin values at baseline and post chemotherapy allow risk stratification regarding impairments in LV function^26^. Because of its prognostic value, it is not surprising that cardiac biomarkers, including troponin, were entered into most of the recently published baseline cardiovascular risk stratification models^27^. In contrast to troponin, it is more obvious that the presence of anaemia predicts impaired exercise capacity due to reduced oxygen supply to skeletal muscles in anaemic patients. Anaemia is an extraordinarily common comorbidity in patients with cancer and its presence is described in 39% at the time of patients’ enrolment and in up to 67% during chemotherapy in the European Cancer Anaemia Survey^28^. Among our patients, anaemia was present in 61.7%, whereby its prevalence was significantly higher in patients with reduced exercise capacity, and multivariable analysis revealed anaemia to be an independent predictor of impaired exercise capacity. The mechanisms of developing anaemia in cancer patients are multiple and are spanning from decreased erythropoiesis to directly destroying blood cells or blood loss itself. In the clinical setting, cancer patients often complain about fatigue, which is a typical sign of anaemia and also often occurs in the context of physical exhaustion^29^. The relation between anaemia and reduced exercise capacity or physical activity is not only described by our group but also is in line with many publications considering especially patients with heart failure^30–33^. With regards to cancer patients, the evidence of an influence of anaemia on exercise capacity has been much less investigated. However, there are no reasons to assume a different relationship in oncological patients. On the other hand, exercise directly affects the number of red blood cells. Drouin et al*.* have shown physical activity to have the potential to stabilise red cell counts and therefore to prevent anaemia in a breast cancer population^34^. This finding is confirmed in a large meta-analysis by Hu and Lin^35^. In patients with chronic heart failure, the presence of anaemia was also found to be an independent predictor of increased mortality^36^. It remains unclear if this result can be translated to cancer patients. Nevertheless, clinicians should consider anaemia to improve patients’ exercise tolerance. If anaemia is the result of iron deficiency, the supply of intravenous iron may help in the correction of anaemia. Additionally, iron deficiency was shown to be a predictor of reduced exercise capacity in patients with chronic heart failure^37,38^. The fact that iron deficiency was not predicting lower exercise capacity in our study could be the result of equal distribution of iron deficiency between patients with lower and better exercise capacity. If iron deficiency is not present and therefore unlikely to be a chief reason for anaemia development, the attending clinician has to choose between blood transfusion treatment and the use of erythropoietin containing drugs. Using the latter imposes an increased risk of thrombosis. To maintain the achieved level of erythrocytes, exercise training should be prescribed as well.

HGS assessment can help to identify patients at high risk of being affected in their exercise capacity. In our analysis, HGS was positively associated with exercise capacity, which is not astonishing because HGS represents the total muscle mass, and a reduced muscle mass may lead to reduced exercise capacity. In agreement with our findings, the relationship between HGS and exercise capacity has already been described for other diseases like COPD and coronary artery disease^39,40^. Furthermore, various studies have documented the correlation between HGS and QoL^41–43^. Cancer patients usually wish to be directly involved in their cancer treatment. Emphasising the meaning of physical activity may give patients therefore the feeling to directly influence the underlying condition. This may be especially important since recent studies were able to illustrate a direct correlation of HGS and cancer mortality^44–46^.

In summary, increasing troponin values are an important predictor of reduced exercise capacity. Due to the ease of their assessment, troponin values should be measured during cancer treatment in order to identify patients at high risk of reductions in exercise capacity. Furthermore, correction of anaemia and assessing HGS may be helpful in maintaining patients’ higher exercise capacity level.

## Limitations

Some limitations need to be addressed. First, the current study included all-comers of a regional cancer centres specialised in the treatment of specific cancers. The all-comer status resulted in different cancer therapeutic strategies, which may have affected the results. Second, the all-comer status implies that our results cannot be directly transferred to a specific cancer type or treatment option. Third, we did not perform analyses of echocardiography parameters and exercise capacity, because patients with relevant cardiovascular diseases (coronary artery disease, prior myocardial infarction, or left ventricular ejection fraction (LVEF) < 50%, atrial fibrillation) were excluded as per study design. Fourth, due to the small sample size some effects may not have reached statistical significance even if there may be a correlation (e.g. present of cachexia and a lower 6MWT). Finally, we decided to define low exercise capacity as a 6-MWT distance below the median of the study cohort, which may have affected the results. However, due to the lack of a universally valid threshold to define low exercise capacity using the median deemed to be most reasonable.

## Data Availability

Data are not publicly available due to ethical and legal issues. The datasets used and/or analyzed during the current study are available from the corresponding author on reasonable request.
